# The long run impact of early childhood deworming on numeracy and literacy: Evidence from Uganda

**DOI:** 10.1371/journal.pntd.0007085

**Published:** 2019-01-31

**Authors:** Kevin Croke, Rifat Atun

**Affiliations:** Department of Global Health and Population, Harvard T.H. Chan School of Public Health, Boston, Massachusetts, United States of America; University of Queensland School of Veterinary Science, AUSTRALIA

## Abstract

**Background:**

Up to 1.45 billion people currently suffer from soil transmitted helminth infection, with the largest burden occurring in Africa and Asia. Safe and cost effective deworming treatment exists, but there is a debate about mass distribution of this treatment in high prevalence settings. While the World Health Organization recommends mass administration of anthelmintic drugs for preschool and school-aged children in high (>20%) prevalence settings, and several long run follow up studies of an influential trial have suggested large benefits that persist over time, recent systematic reviews have called this recommendation into question.

**Methods and findings:**

This paper analyzes the long-term impact of a cluster-randomized trial in eastern Uganda that provided mass deworming treatment to preschool aged children from 2000 to 2003 on the numeracy and literacy skills of children and young adults living in those villages in 2010-2015. This study uses numeracy and literacy data collected seven to twelve years after the end of the deworming trial in a randomly selected subset of communities from the original trial, by an education-focused survey that had no relationship to the deworming study. Building on an earlier working paper which used data from 2010 and 2011 survey rounds, this paper uses an additional four years of numeracy and literacy data (2012, 2013, 2014, and 2015). Aggregating data from all survey rounds, the difference between numeracy scores in treatment versus control communities is 0.07 standard deviations (SD) (95% CI -0.10, 0.24, p = 0.40), the difference in literacy scores is 0.05 SD (95% CI -0.16, 0.27, p = 0.62), and the difference in total scores is 0.07 SD (95% CI -0.11, 0.25, p = 0.44). There are significant differences in program impact by gender, with numeracy and literacy differentially positively affected for girls, and by age, with treatment effects larger for the primary school aged subsample. There are also significant treatment interactions for those living in households with more treatment-eligible children. There is no evidence of differential treatment effects on age at program eligibility or number of years of program eligibility.

**Conclusions:**

Mass deworming of preschool aged children in high prevalence communities in Uganda resulted in no statistically significant gains in numeracy or literacy 7-12 years after program completion. Point estimates were positive but imprecise; the study lacked sufficient power to rule out substantial positive effects or more modest negative effects. However, there is suggestive evidence that deworming was relatively more beneficial for girls, primary school aged children, and children living in households with other treated children.

**Research approval:**

As this analysis was conducted on secondary data which is publicly available, no research approval was sought or received. All individual records were anonymized by the data provider prior to public release.

## Author summary

Mass deworming is recommended by the World Health Organization for health benefits to communities where soil-transmitted helminth infection is endemic. In addition to health benefits, several recent studies find long run educational or economic benefits for cohorts dewormed as children. In this paper, treatment and control communities which formed part of a cluster randomized deworming trial in eastern Uganda from 2000-2003 were surveyed from 2010-2015 to measure children’s basic numeracy and literacy. We analyze this data to see if there are detectable improvements in basic academic skills among children in the dewormed communities. We find that mass deworming of preschool aged children in high prevalence communities in Uganda resulted in no statistically significant gains in numeracy or literacy 7-12 years after program completion. However, there is suggestive evidence that deworming is relatively more effective for girls, primary school aged children, and children living in households with other treatment-eligible children.

## Introduction

As many as 1.45 billion people are estimated to suffer from intestinal worms (also known as soil-transmitted helminths, STH) such as roundworm, hookworm, or whipworm, largely in sub-Saharan Africa and in South Asia [1]. Worm infections are primarily transmitted through fecal-oral contact, and although rarely fatal, moderate to heavy infections are associated with a wide range of health problems including stunted growth, reduced nutrient absorption, and anemia [2, 3]. Among pre-school age children, worm infection is associated with slowed growth, and among school age children it has been linked to poor attendance at school and reduced performance on cognitive tasks [4]. Inexpensive, safe and effective treatment is available, in the form of drugs such as albendazole or mebendazole.

The existence of inexpensive treatment that has no significant side effects for uninfected children, and the fact that screening children for infection is significantly more expensive than treating them, has led the World Health Organization to advocate annual deworming of all children and women of child-bearing age in regions where STH prevalence is over 20%, and twice annually where prevalence is above 50%. Several recent studies have found large benefits to such deworming interventions in high prevalence settings, with some showing educational benefits (such as increased school attendance or improved performance on cognitive tests) [5, 6] while others demonstrate improved long run adult economic outcomes [7]. The evidence from these studies, and from earlier biomedical trials, has led various international expert groups to rank mass deworming as a highly cost-effective intervention in endemic areas [8, 9, 10]. These findings from the economics literature, together with the WHO recommendations, appear to be at odds with the conclusions of a series of Cochrane Collaboration and Campbell systematic reviews, which find no evidence of the benefits of mass administration of deworming drugs [11, 12]. Several authors from the Cochrane review argue that “the time has come for donors, governments, and philanthropists to call it a day” on mass deworming [13]. By contrast, a different review of the meta-analysis literature found that mass deworming does have effects on weight gain, estimating average weight gain of 0.13 kg (95% CI 0.03, 0.24); and weight gain of 0.18 kg (95% CI 0.07, 0.29) in settings where worm prevalence is above 50% [14].

However, while the debate continues about the short run health effects of deworming, other actors have been more interested in the potential long run benefits of deworming [15], largely as a result of several experimental studies that find positive long run effects of a magnitude that would make deworming an extremely cost-effective intervention [6, 7]. This paper contributes to this literature by examining the long run effects of a cluster-randomized mass deworming trial targeting preschool aged children (aged one to seven years) in 48 villages between 2000 and 2003 in eastern Uganda [16]. A working paper which used educational data to estimate the preliminary long run impact of this deworming was released in 2014, showing large and statistically significant gains for the treatment group (0.30-0.36 SD increases in numeracy compared to controls). This working paper used data from the 2010 and 2011 survey rounds of the annual Uwezo education survey [17]. Since then, 2012, 2013, 2014, and 2015 data was released by Uwezo. This paper therefore subsumes the 2014 working paper by incorporating an additional four years of data on this population.

### Literature: Deworming and cognitive or educational outcomes

The theory that deworming could improve educational outcomes goes back at least as far as the 1930s [18], but there was little rigorous research on the topic until a series of trials in the 1990s testing the impact of deworming on cognitive outcomes over short periods (1-7 months) [19, 20, 21], followed by a series of mass treatment trials which recorded educational or cognitive outcomes. The Cochrane and Campbell reviews [11, 12] identify a relatively modest number of multiple dose mass deworming trials with cognition outcomes. Both reviews note their limited ability to aggregate these trials due to incomparable outcome measures, but find that there is no evidence of positive effect from deworming on these outcomes. However, these trials largely studied school age children, while the deworming program examined in this paper targeted pre-school aged children. There is a priori reason to believe that improved early childhood nutrition can have larger effects on a range of outcomes, including cognition, than improved nutrition for school aged children [22, 23, 24]. Going back to the Barker hypothesis [25], evidence has accumulated that early child nutritional deprivation, such as from famine, has long run consequences for long run health, well-being, and cognition [26, 27, 28, 29]. This implies that interventions that mitigate early childhood nutritional deficits could have long run benefits [24, 30, 31]. Another theme is the broad range of shocks that appear to have important effects, including some seemingly mild shocks [32]. For this age group, the findings from the deworming meta-analysis literature are sparser. A smaller group of studies in the Campbell review examine cognitive outcomes for preschool aged children [33, 34]. Neither finds a significant benefit, although both papers note that the studies in question were not powered for cognitive outcomes.

Evidence of short run benefit on education-related outcomes from deworming of school-aged children comes from Miguel and Kremer [5], who use a cluster randomized design to show increases in school attendance resulting from deworming. In addition, they identify spillovers both within treatment schools, and at schools up to 3 km away from treatment schools. Replication of this paper and subsequent exchanges with Aiken et al. [35] confirmed their finding of within-school spillovers and geographic spillovers up to 3 km, but showed that spillover benefits did not extend beyond 3 km range as the original paper argued. High levels of STH prevalence in this setting could explain the benefits; by contrast a recent trial in a low prevalence setting did not find comparable educational gains [36].

Evidence for longer run benefits of deworming comes from follow up data collection on the cohorts from the Miguel and Kremer study. Baird et al. [7] find marked gender-related patterns to gains in their long run follow up of the original trial: while men see employment related gains, they do not gain more education. By contrast, women are one third more likely to attend secondary school, and have improved self-reported health and increased body mass index.

Ozier [6] examines whether younger siblings of children treated in the original Miguel and Kremer trial show cognitive benefit, conditional on the age at which they were exposed to the spillover. Children whose communities were treated before they were 1 year old show large benefit (via spillovers from sibling treatment), while children treated after that age show no evidence of benefit. Furthermore, the effects were twice as large for those likely to have had treated older siblings in their household, suggesting that children may benefit more from deworming when their siblings are also dewormed.

## Methods

This study examines the long run impact of a World Bank-funded cluster randomized deworming trial on 27,995 children in 48 parishes (communities) in 5 districts in Eastern Uganda from November 2000 to June 2003. (Originally 50 parishes were selected but two dropped out of the trial. We conduct intention-to-treat analysis based on these original 50 communities). These districts were targeted for the intervention because a parasitological survey showed that over 50% of children between ages 5 and 10 were infected with intestinal worms, most often hookworm [37]. The intervention was delivered via a Child Health Day (CHD). Child Health Days are public events, held biannually, in which all parents in a given catchment area are requested to bring pre-school age children to a treatment site to receive a set of basic health services such as Vitamin A supplementation, growth monitoring, and vaccines. At the five Child Health Days held over the course of the project, children in the control parishes who attended the event were offered the standard CHD interventions, while those in the treatment group were also given deworming treatment (400 mg albendazole). All children between age 1 and 7 at the sessions (except those who were ill at the time) were given albendazole in the treatment group, while none were offered it in the control group. This recruitment procedure generated balance between the treatment and control group on baseline nutrition indicators (i.e. percentage of underweight children) as reported in the initial trial. Randomization was at the administrative level immediately above the village, known as the “parish” in Uganda. A short run evaluation of the program found evidence of weight gain for the treatment group in adjusted models [16].

### Power calculations

With 36 clusters and an intra-cluster correlation (ICC) of 0.02 for numeracy scores, we calculate an intention-to-treat minimum detectable effect of 0.18 standard deviations (for the numeracy outcome) if all children were treated in the treatment group and none were treated in the control group (the ICC is calculated from Uwezo data in study clusters, and the power calculation is implemented using Stata’s “clustersampsi” command). Inflating this to account for incomplete take up in the treatment group (74% of the treatment group reported attending Child Heath Days) implies a minimum detectable effect (MDE) for numeracy of 0.33 standard deviations (SD). (Some control group deworming was reported from private drug shops. However without information about the frequency or intensity of this deworming we cannot precisely adjust power calculations to reflect it).

### Ethical considerations

As this analysis was conducted on secondary data which is publicly available, no research approval was sought or received. All individual records were anonymized by the data provider prior to public release.

### Data

Outcome data in this study comes from the Uwezo initiative, which conducts large-scale annual surveys to test basic literacy and numeracy in Kenya, Tanzania, and Uganda. Every year, Uwezo randomly samples 30 villages in every district in all three countries, and all children between ages 6 and 16 in 20 households per village are tested on basic literacy and numeracy [38]. Further details about Uwezo’s sampling procedures are available in Supporting Information Section 1.

In the Uwezo survey, children are given a numeracy test with seven skill areas of ascending difficulty, and a literacy test with six skill areas, for a maximum of 13 points. Following standard practice in the education literature, standardized math, literacy, and total (math plus literacy) scores are created, with mean zero and standard deviation of 1, as outcome variables.

In all rounds, the basic math test consists of seven categories, representing counting objects up to 10, number recognition of numbers from 10-999, addition, subtraction, multiplication, and division. There are bonus questions across all years but since these vary in content substantially (from practical questions about buying items in market in some years, to place value questions in other years), we omit them. The literacy test includes five categories, from letter recognition, to the ability to read words, sentences, and paragraphs. There are two comprehension questions, for which one point is awarded, in early rounds, while in later survey rounds two points are awarded for the two questions. Since the meaning of a comprehension bonus point appears to change over time (and is missing for most respondents in 2010), we omit the bonus comprehension questions for all rounds and simply use the 5 point literacy scale which remains fundamentally unchanged across all six survey rounds.

### Statistical methods

The Uwezo survey captures numeracy and literacy outcomes for a small fraction of the population exposed to the original program (the Uwezo sample is 2,210 while there were 27,995 individuals in the original trial). However, Uwezo’s sampling method makes it likely that the 2,210 individuals surveyed are in effect a random subsample of individuals from the communities in the original sample. This is because according to its procedures, the Uwezo survey sampled a random subset of villages from the original trial districts, and a random group of households from within these villages.

Exploiting the random allocation of treatment in the original trial, and the random sampling of a subset of original trial communities by Uwezo, the effect of the deworming program is estimated by regressing test scores on a treatment indicator variable (unadjusted model) and on the treatment variable together with dummy variable controls for each age category (ages 6 through 16), each survey round (2010, 2011, 2012, 2013, 2014, and 2015), and gender, and all interactions of gender, age, and survey round (adjusted model).

The specifications are:

$$\begin{array}{c} y_{ic}=\beta_{0}+\beta_{1}treat_{c}+\varepsilon_{i,c} \end{array}$$ (1)

for unadjusted specifications (odd numbered columns), and for adjusted specifications (presented in even numbered columns),

$$\begin{array}{c} y_{ic}=\beta_{0}+\beta_{1}treat_{c}+\chi_{ic}^{\prime }\cdot \gamma +\varepsilon_{i,c} \end{array}$$ (2)

where *i* is for example an individual and *c* is a community (parish). *y*_*i*,*c*_ is the learning outcome of interest, *treat*_*c*_ is a dummy for treatment communities from the original trial, and $\chi_{i}^{\prime }$ is a vector of indicator variables for each age, gender, and survey round, and all interactions of these age, gender, and survey round indicators. The coefficient of interest is *β*_1_. *ε*_*i*,*c*_ is the error term, clustered at the parish level. Based on reported age, a child’s age in the years of 2000, 2001, 2002, and 2003 is calculated. If the child was between age 1 and age 7 during any one of those years, he or she is considered to be in the age group potentially exposed to the program.

Motivated by the broader deworming literature, we also examine potential modifiers of effect. We seek to do so in ways which are robust to concerns about multiple hypothesis testing. Therefore we first conduct the main analysis, and the heterogeneity analysis, using the specifications used in the 2014 working paper which was mentioned previously. In effect we treat this 2014 working paper as analogous to a pre-registered analytical plan for the full paper.

Then, in more exploratory vein, we examine additional hypotheses which we believe are appropriate given changes in the sample across rounds, as well as hypotheses which have been generated by other recent papers in the literature. We examine the following dimensions of heterogeneity on the treatment effect of deworming: primary school vs. secondary school age, potential exposure to other dewormed children, and age at first eligibility for the program. Given the exploratory nature of these analyses, we calculate q-values for the coefficients of interest, limiting the false discovery rate using the method proposed by Benjamini and Hochberg [39]. (We also examine these effect modifiers on school enrolment and attendance in Supporting Information S5 Table).

For hetereogeneity analysis, we interact the treatment variable with variables created to represent poverty, gender, and cumulative years of program eligibility (for the pre-specified analyses), and age at program initiation, primary school age vs. post-primary school age, and number of other deworming-eligible children in the household (for the exploratory analyses). As a proxy for poverty, we calculate the first principal component of a wealth index made up of the household asset variables which are measured consistently across all survey rounds (access to electricity, and ownership of a television, radio, and phone), and create a “low assets” indicator which represents the bottom two wealth quintiles. To represent households with multiple treatment-eligible children we create a variable which represents the number of other treatment-eligible children in the household (excluding the respondent him or herself). The “years of program eligibility” variable is calculated by summing up the number of years that a respondent was of program-eligible age (age 1 to age 7) between 2000-2003, and creating a binary variable equal to one for respondents who were eligible for 2, 3, or 4 years of the program. The primary school age variable is binary, equal to one for respondents aged 7-13 at the time of survey, and equal to 0 for respondents aged 14-16. The “age at first eligibility” variable is a binary variable equal to 1 for respondents who were age 1 when the program was initiated (in 2000), and equals 0 for those who were older than age 1 in when the program was first initiated.

We also conduct a range of robustness checks for the main results. Since survey non-response could bias results, we re-run the main analysis using predicted scores (for respondents who did not take the Uwezo test) generated by the Uwezo team based on observable respondent characteristics [40], as well as using inverse probability weighting to minimize any bias due to non-response. In addition, we use a different dataset, the Uganda National Panel Survey, to examine whether migration out of the study sample is correlated with being of post-primary school age, and with literacy. We also conduct all additional robustness checks which were implemented in the 2014 working paper. Finally, we also run the main analysis using population weights provided by Uwezo. Since Uwezo conducted stratified sampling (with equal numbers of communities sampled from both densely and thinly population districts), observations can be re-weighted to be more representative of the population [40]. All of these robustness checks are presented in the Supporting Information.

## Results

Table 1 presents features of the sample. There were five survey rounds from 2010–2015. Two rounds (2010 and 2014) did not sample every district of Uganda, and accordingly sampled relatively few of the original trial communities (2 and 7 clusters respectively). The 2011, 2012, 2013, and 2015 survey rounds were full size surveys which sampled from every district in Uganda, including all 5 districts from the original trial. As a result they sampled between 17 and 21 of the original trial communities per round. In total, 36 out of the original 50 trial communities were sampled by Uwezo; 18 treatment and 18 control. 2,210 out of the 4,154 children sampled in the original trial communities were between the ages of 1 and 7 during the deworming trial period, and were therefore potentially exposed to the program. Out of these 2,210 respondents, there were missing values for 7.2% of math (158 out of 2,210) and 7.1% of literacy scores (157 out of 2,210). Thus there are 2,052 individuals in the final math sample and 2,053 in the final English sample. There is no differential non-response associated with treatment, although age is highly correlated with non-response (see Supporting Information, S3 Table).

**Table 1:** Covariate balance between treatment and control individuals, 2010-2015.

Variable	Observations	control %	control N	treatment %	treatment N	P-value
Age	2210	12.9	1174/2210	12.9	1036/2210	0.82
Female	2210	0.47	1174/2210	0.51	1036/2210	0.15
Owns phone	2210	0.56	1174/2210	0.55	1036/2210	0.91
Owns radio	2210	0.63	1174/2210	0.59	1036/2210	0.35
Owns television	2210	0.07	1174/2210	0.06	1036/2210	0.71
Has electricity	2210	0.06	1174/2210	0.05	1036/2210	0.89
Direct access to water	2210	0.22	1174/2173	0.30	1036/217	0.29
Female headed household	2210	0.22	1174/2210	0.24	1036/2210	0.74
mother post-primary	2047	0.10	1062/2047	0.09	987/2047	0.96
Child attends private school	2137	0.15	1140/2137	0.15	997/2137	0.88
p value of F test						0.85

*Notes*: P values are from regressions of each individual variable on treatment status, with robust standard errors clustered at parish level. The F test is from a regression with treatment as the dependent variable and all of the listed variables as covariates. Mother’s age was included in the original working paper balance tables; it had to be excluded here because the question was omitted from later survey rounds. Sample is restricted to program-exposed age cohorts (aged 1-7 from 2000 to 2003). * *p* < 0.1, ** *p* < 0.05, *** *p* < 0.01.

### Main results

Across the three outcome variables, there is no statistically significant treatment effect of deworming in the full sample. In adjusted specifications, the coefficients for numeracy (0.07 SD, standard error (s.e.) 0.08), literacy (0.05 SD, s.e. 0.11) and total score (0.07 SD, s.e. 0.09) are not significantly different from zero (Table 2).

**Table 2:** Effect of deworming program on test scores.

	numeracy		literacy		total	
	(1)	(2)	(3)	(4)	(5)	(6)
treat	0.0535 (0.0800)	0.0697 (0.0818)	0.0409 (0.113)	0.0527 (0.105)	0.0512 (0.0919)	0.0685 (0.0879)
controls included?	no	yes	no	yes	no	yes
*N*	2052	2052	2053	2053	2031	2031
*R*^2^	0.001	0.231	0.000	0.245	0.001	0.273

Robust standard errors clustered at parish level in parentheses. Controls (in columns 2, 4, and 6) include gender, age, and survey round, and all interactions of these variables.

* p< .1, ** p< .05, *** p< .01

The other educational outcomes captured in the Uwezo dataset relate to whether the child is currently in school, and whether he or she ever enrolled in school. There is no significant relationship between deworming program exposure and attendance or school enrollment (Table 3); the coefficients in adjusted specifications on being in school at the time of the survey or never having enrolled are -0.01 (s.e. 0.01) and 0.01 (s.e. 0.01).

**Table 3:** School attendance and enrollment.

	in school		never enrolled	
	(1)	(2)	(3)	(4)
treat	-0.0100 (0.0103)	-0.0141 (0.0104)	0.00710 (0.00462)	0.00694 (0.00471)
controls included?	no	yes	no	yes
*N*	2210	2210	2210	2210
*R*^2^	0.000881	0.0545	0.00214	0.0230

*Notes*: Linear probability model (OLS). Robust standard errors clustered at parish level in parentheses. Controls (in columns 2 and 4) are gender, survey round, and age fixed effects, and all interactions of these variables. * *p* < 0.1, ** *p* < 0.05, *** *p* < 0.01.

### Heterogeneity analysis

In this section we focus on several dimensions of heterogeneity in treatment effect which were examined in the 2014 working paper: gender, poverty (proxied by asset ownership), and number of years eligible for treatment, as well as three additional dimensions of heterogeneity (presence of other program-eligible children in the household, primary vs. post-primary school age, and age at first program eligibility), which are exploratory in nature.

#### Gender

There are two notable gender-related patterns with regard to learning outcomes. First there are significant differences in numeracy by gender, with girls benefiting significantly more than boys from deworming: the interaction terms for treatment by gender for adjusted numeracy, literacy and total scores are 0.149 SD (s.e. 0.0798) for numeracy, 0.148 SD (s.e. 0.0792) for literacy, and 0.160 SD (s.e. 0.0777) for total scores; p values range from 0.04 to 0.07. Second, while the treatment effects in a model which restricts the sample to girls only are not significant, the effects are substantively large (0.13–0.16 SD), and approach marginal significance for numeracy, with p values of 0.13-0.14 (Table 4).

**Table 4:** Gender interaction for learning outcomes.

	numeracy		literacy		total	
	(1)	(2)	(3)	(4)	(5)	(6)
**Panel A: treatment effects by gender**						
treat × female	0.196* (0.0974)	0.149* (0.0798)	0.215** (0.104)	0.148* (0.0792)	0.217** (0.0999)	0.160** (0.0777)
treat	-0.0429 (0.0871)	-0.00411 (0.0858)	-0.0678 (0.120)	-0.0209 (0.108)	-0.0562 (0.0946)	-0.0104 (0.0866)
female	-0.112 (0.0813)	-0.561 (0.444)	-0.0250 (0.0948)	0.136 (0.168)	-0.0801 (0.0881)	-0.304 (0.231)
controls included?	no	yes	no	yes	no	yes
*N*	2052	2052	2053	2053	2031	2031
*R*^2^	0.00405	0.232	0.00481	0.246	0.00447	0.274
**Panel B: female only**						
treat	0.154 (0.0995)	0.145 (0.0954)	0.147 (0.130)	0.127 (0.117)	0.160 (0.114)	0.149 (0.105)
controls included?	no	yes	no	yes	no	yes
*N*	1010	1010	1012	1012	998	998
*R*^2^	0.00752	0.236	0.00547	0.261	0.00788	0.284

In Panel A, “treat × female” represents the differential effect of treatment for female respondents compared to male respondents. The uninteracted treatment coefficient presents treatment effects for male respondents. Controls (in columns 2, 4, and 6) include gender, age, and survey round, and all interactions of these variables. Robust standard errors clustered at parish level in parentheses.

* p< .1,

** p< .05,

*** p< .01

#### Poverty

When interacting the low assets indicator with treatment, there is some evidence of a differential effect for poorer quintiles. The coefficient on treatment, and the coefficient on low assets times treatment, are of roughly comparable magnitudes but opposite signs, suggesting a positive effect for the non-poor and an approximately zero (or modestly negative) effect for the poor (Table 5).

**Table 5:** Poverty/Low assets interaction.

	numeracy		literacy		total	
	(1)	(2)	(3)	(4)	(5)	(6)
treat × low assets	-0.172* (0.0943)	-0.178* (0.0919)	-0.144 (0.108)	-0.137 (0.0985)	-0.174* (0.0989)	-0.170* (0.0929)
treat	0.127 (0.0924)	0.148 (0.0956)	0.101 (0.136)	0.113 (0.123)	0.124 (0.106)	0.143 (0.100)
low assets	-0.0236 (0.0783)	-0.0496 (0.0719)	-0.135 (0.0924)	-0.180** (0.0845)	-0.0686 (0.0869)	-0.106 (0.0791)
controls included?	no	yes	no	yes	no	yes
*N*	2052	2052	2053	2053	2031	2031
*R*^2^	0.00669	0.238	0.0119	0.260	0.00975	0.284

The “treat × low assets” coefficient represents the differential effect of treatment for respondents in low asset households, compared to respondents in non-low asset households. The uninteracted treatment coefficient represents treatment effects for respondents in non-low asset households. “Low assets” denotes households in the bottom two wealth quintiles. Wealth quintiles are created based on the first principal component of a household wealth index incorporating the household asset variables which are measured consistently across all survey rounds (access to electricity, and ownership of a television, radio, and phone). Controls (in columns 2, 4, and 6) include gender, age, and survey round, and all interactions of these variables. Robust standard errors are clustered at parish level in parentheses.

* p< .1,

** p< .05,

*** p< .01

#### Years of program eligibility

We also test whether effects differ by intensity of potential program exposure, as measured by the number of years of treatment that children were eligible for. Due to variation in children’s age at the time of program initiation, there are several age cohorts which were only eligible for 0 or 1 year of treatment, compared to cohorts eligible for 2, 3 or 4 years. We find no significant additional benefit to additional years of treatment eligibility, using a specification which interacts treatment with an indicator for whether respondents are eligible for 2-4 years of treatment (Table 6).

**Table 6:** Treatment intensity interaction.

	numeracy		literacy		total	
	(1)	(2)	(3)	(4)	(5)	(6)
treat × 2–4 years exposure	0.000999 (0.0792)	0.000397 (0.0740)	0.0275 (0.0905)	0.0493 (0.0784)	0.0134 (0.0844)	0.0217 (0.0764)
treat	0.0328 (0.0592)	0.0714 (0.0508)	0.000923 (0.0745)	0.0153 (0.0700)	0.0206 (0.0564)	0.0541 (0.0485)
2-4 years exposure	1.093*** (0.0543)	-0.1364 (0.2009)	1.044*** (0.0513)	-0.417** (0.187)	1.145*** (0.0536)	-0.262 (0.200)
controls included?	no	yes	no	yes	no	yes
*N*	3850	3850	3851	3851	3795	3795
*R*^2^	0.296	0.459	0.276	0.425	0.328	0.497

The “treat × 2–4 years exposure” coefficient represents the differential effect of treatment for respondents exposed to treatment for 2-4 years, compared to the effect for those exposed for less than 2 years. The uninteracted treatment coefficient presents treatment effects for respondents who were exposed to treatment for less than 2 years. Additional controls (in columns 2, 4, and 6) include gender, age, and survey round, and all interactions of these variables.

* p< .1,

** p< .05,

*** p< .01

#### Pre-secondary school vs. secondary school age groups

We analyze whether treatment effects are larger for respondents who were of primary school age (defined as age 6-13) at the time of their interview compared to those of secondary school age (age 14-16). This specification is motivated by potentially selective migration out of the sample by respondents of post-primary school age.

Primary schooling in Uganda starts officially at age 6, and lasts seven years, so a child who started the first year of formal schooling (Primary 1) at age 6 1/2 and completed all seven years without repetition would complete Primary 7 at age 13 1/2, and would start secondary school at age 13 or 14.

While treatment effects for the under 14 subsample alone fall short of significance (0.16 SD, s.e. 0.10, p = 0.12 adjusted, for numeracy), when interacting treatment with an indicator for being aged under 14 in the full sample (Table 7), we see large positive interaction effects: The treatment for those under 14 is 0.23 SD greater than the treatment effect for those aged 15 or 16 for numeracy (s.e. 0.07, p = 0.003, q = 0.018 adjusted), 0.15 SD greater for literacy (s.e. 0.08, p = 0.07, q = 0.12 adjusted), and 0.21 SD greater for the total score (s.e. 0.07, p = 0.006, q = 0.027 adjusted).

**Table 7:** Primary school age (under 14) interaction.

	numeracy		literacy		total	
	(1)	(2)	(3)	(4)	(5)	(6)
**Panel A: treatment × under age 14**						
treat × under 14	0.208** (0.0783)	0.231*** (0.0735)	0.0992 (0.0991)	0.149* (0.0795)	0.172** (0.0822)	0.206*** (0.0700)
treat	-0.0672 (0.0591)	-0.0680 (0.0632)	-0.0115 (0.111)	-0.0361 (0.0985)	-0.0458 (0.0682)	-0.0542 (0.0649)
under14	-0.713*** (0.0551)	-2.177*** (0.529)	-0.792*** (0.0747)	-1.797*** (0.252)	-0.796*** (0.0607)	-2.152*** (0.443)
controls included?	no	yes	no	yes	no	yes
*N*	2052	2052	2053	2053	2031	2031
*R*^2^	0.119	0.235	0.135	0.246	0.151	0.276
p-value of interaction term	0.012	0.003	0.324	0.069	0.044	0.006
q-value of interaction term	0.043	0.018	0.486	0.124	0.088	0.027
**Panel B: under age 14 subsample**						
treat	0.141 (0.100)	0.163 (0.103)	0.0877 (0.127)	0.113 (0.121)	0.126 (0.113)	0.151 (0.110)
controls included?	no	yes	no	yes	no	yes
*N*	1216	1216	1216	1216	1205	1205
*R*^2^	0.00579	0.168	0.00208	0.155	0.00470	0.189

In Panel A, “treat × under 14” represents the differential effect of treatment for respondents under 14, compared to the effect for respondents over 14. The uninteracted treatment coefficient presents treatment effects for respondents over age 14. Additional controls (in columns 2, 4, and 6) include gender, age, and survey round, and all interactions of these variables. Robust standard errors clustered at parish level.

* p< .1,

** p< .05,

*** p< .01

**Fig 1:**
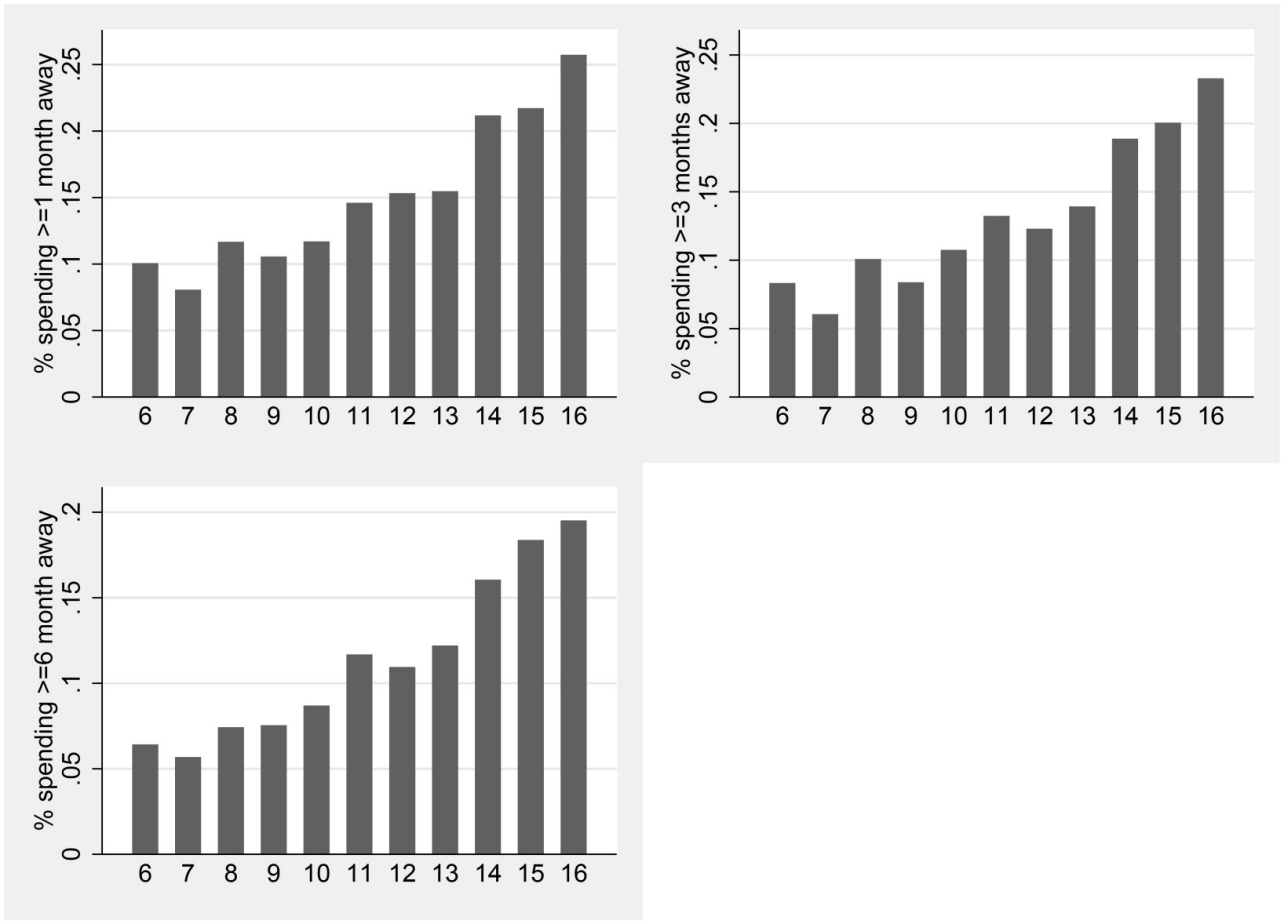
Children’s migration by age. Percentage of respondents who have spent at least 1, 3 or 6 months away from home over the last 12 months. Source: Uganda National Panel Survey. 2009-2010.

Using the Uganda National Panel Survey data, Fig 1 shows that migration is much more common in the over age 14 subsample than in the age 6-13 subsample: 23% of 14-16 year olds had been absent from the household for at least 1 month over the past year, compared to 12% of 6-13 year olds. Supporting Information S4 Table shows that literacy is positively associated with migrating, controlling for age, poverty, gender, rural location, relationship to household head, and district fixed effects.

#### Intra-household spillovers

In this section we focus on potential intra-households spillovers of deworming treatment, by testing whether benefits are larger for respondents who, in addition to being treatment-eligible themselves, also had siblings eligible for treatment. Since worms are spread via worm eggs which are excreted from one person and then reinfect another, children living in households with many other children could experience frequent infection and reinfection by their siblings. (22% of the sample are the only children in their household who were eligible for treatment, while 78% lived with other treatment eligible children). This interaction with treatment with number of other children eligible for treatment is significant, with p<0.05 for all three outcomes and q<0.05 for numeracy and total scores (adjusted) (Table 8).

**Table 8:** Within household spillover interaction.

	numeracy		literacy		total	
	(1)	(2)	(3)	(4)	(5)	(6)
treat × others treated	0.0862** (0.0338)	0.109*** (0.0318)	0.0600 (0.0484)	0.0815** (0.0354)	0.0792** (0.0327)	0.104*** (0.0254)
treat	-0.0756 (0.0872)	-0.0946 (0.0786)	-0.0503 (0.120)	-0.0710 (0.0959)	-0.0680 (0.0938)	-0.0889 (0.0780)
others treated	-0.0479* (0.0278)	-0.0501** (0.0236)	-0.0227 (0.0213)	-0.0345* (0.0196)	-0.0407* (0.0236)	-0.0488** (0.0208)
controls included?	no	yes	no	yes	no	yes
*N*	2052	2052	2053	2053	2031	2031
*R*^2^	0.00464	0.236	0.00190	0.247	0.00374	0.278
p-value of interaction term	0.015	0.002	0.223	0.027	0.021	0.000
q-value of interaction term	0.045	0.018	0.365	0.061	0.054	0.000

The “treat × others treated” coefficient represents the differential effect of treatment for respondents according to the number of other children exposed to treatment, compared to the effect for households with no other children exposed to treatment. Treatment is interacted with the continuous “number of other children exposed in household” variable. The uninteracted treatment coefficient presents treatment effects for respondents who were the only child exposed to treatment in their household. Controls (in columns 2, 4, and 6) include gender, age, and survey round, and all interactions of these variables. Robust standard errors clustered at parish level.

* p< .1,

** p< .05,

*** p< .01

#### Age at program eligibility

In this section we examine whether the treatment is larger for cohorts potentially exposed the program early in infancy, compared to those potentially exposed at older ages (Two-thirds of the sample was first eligible for the program at age 1). We interact treatment with program exposure at age 1, controlling separately for number of years of program exposure, and see no significant interaction between treatment and early age of treatment initiation (Table 9).

**Table 9:** Early exposure (at age 1) treatment interaction.

	numeracy		literacy		total	
	(1)	(2)	(3)	(4)	(5)	(6)
treat × program eligible at age 1	0.0345 (0.0735)	0.0546 (0.0697)	0.0232 (0.0937)	0.0333 (0.0895)	0.0248 (0.0789)	0.0374 (0.0733)
program eligible at age 1	-0.231*** (0.0548)	0.441* (0.229)	-0.363*** (0.0769)	1.416*** (0.379)	-0.298*** (0.0597)	0.838*** (0.251)
treat	0.00871 (0.0895)	0.0327 (0.0869)	0.000840 (0.137)	0.0301 (0.121)	0.0118 (0.107)	0.0431 (0.0952)
controls included?	no	yes	no	yes	no	yes
*N*	2052	2052	2053	2053	2031	2031
*R*^2^	0.116	0.231	0.135	0.245	0.150	0.273
p-value of interaction term	0.642	0.439	0.806	0.712	0.755	0.614
q-value of interaction term	0.770	0.608	0.806	0.799	0.799	0.770

The “treat × program eligible at age 1” coefficient represents the differential effect of treatment for respondents who were program eligible at age 1, compared to the effect for respondents who were eligible after age 1. The uninteracted treatment coefficient presents treatment effects for respondents who were eligible after age 1. Additional controls (in columns 2, 4, and 6) include gender, age, and survey round, and all interactions of these variables. Robust standard errors clustered at parish level.

* p< .1,

** p< .05,

*** p< .01

## Discussion

This study addresses the question of whether a mass deworming program in a high prevalence setting had a measurable impact on basic academic skills, such as numeracy and literacy, for children who live in villages which were part of this program during their preschool years. While an earlier working paper using the 2010 and 2011 Uwezo data suggested that deworming can have important long run impact on these skills, analysis incorporating all data from 2010–2015 shows that there is no statistically significant effect of early childhood deworming on numeracy or literacy 7-12 years later.

However, there is suggestive evidence of differential impact along several dimensions. We find that the program differentially affected female respondents, and that treatment effects for females alone, although imprecisely estimated and not statistically significant at conventional levels, are substantively large. In exploratory heterogeneity analysis, we note that treatment effects for the primary school age (7-13 year old) respondents are significantly larger than for the post-primary (14-16 year old) cohorts. In addition, motivated by recent findings on spillovers in deworming programs, we find that the treatment effect increases as the number of treatment-eligible children in a household increases. However, we do not find evidence that initiation of program eligibility at age 1 results in larger effects than initiation after age 1, and we do not find evidence of differential gains for age cohorts who had more years of program eligibility.

What factors might explain the difference between the findings of the original working paper and this analysis?

A published critique of the 2014 working paper [41] criticized it for only having information about a subsample of original trial communities, and for potential imbalance. However, as noted by several participants in 2016 *International Journal of Epidemiology* symposium about this and related papers, the Uwezo sampling procedure leaves no reason to expect imbalance in which of the trial communities were sampled by the Uwezo survey [42, 43, 44], and the full sample demonstrates no significant imbalance between the treatment and control groups across a broad range of covariates. There are no statistically significant differences on any of the variables tested, and an F test of a regression of all the variables in the table below on treatment has a p value of 0.85.

A potential explanation is suggested by the heterogeneity analysis, which showed larger effects for primary school aged children. A key difference between this paper and the original working paper is the age profile of the sample. Compared to the sample used in the original working paper, the 2010-2015 sample is now more heavily composed of post-primary school age cohorts. In the first three years of the sample (2010-2012), 34% of the sample were aged 14-16, compared to 54% in the 2013-2015 surveys. If 14, 15, and 16 years olds are more likely to migrate away from their home community than primary school aged children, it is more likely that they are interviewed in a community in which they did not live at the time the program was implemented (2000-2003). This introduces measurement error into the independent variable (potential exposure to the deworming program in early childhood), and therefore biases any potential treatment effects towards zero [45]. It could also bias effects downward if more literate and numerate individuals have higher propensity to leave their home communities.

Differential migration rates by age and skill are impossible to document in the Uwezo sample, since Uwezo household rosters only include children and young adults living at home; those who have migrated are simply not observed in the data. However, using the 2009-2010 Uganda National Panel Survey (which includes both household members living both at home, as well as those living outside the household for education or work purposes), we document differential migration among the aged 14-16 cohorts, and among the more literate. This is consistent which evidence from other developing countries: For example, Young [46] aggregates DHS data from 65 developing countries to shows that education is correlated with rural-urban migration, while Hicks et al. [47] use panel surveys in Kenya and Indonesia to show that cognitive ability is strongly associated with rural-urban migration, even after adjusting for education. In Uganda, Mensah and O’Sullivan [48] show associations between education and migration in the Uganda National Panel Survey.

A weakness of this study is that we do not know specifically that the children and young adults surveyed by Uwezo were directly exposed to the deworming program. We do know that over 70% of children in treatment communities attended Child Health Days and were dewormed, but some unknown fraction of the respondents may have been born in different communities than the ones they were surveyed in, or else did live in the communities but never attended the deworming events.

Another limitation of the study is that, after the trial from 2000-2003, the government of Uganda incorporated deworming into Child Health Days, meaning that some percentage of children in the control group received some deworming treatments after the original trial period concluded [49]. This would attenuate any benefits experienced by children in the original treatment group, since both treatment and control communities were potentially exposed to national deworming programs after 2003.

How should this pattern of results be interpreted, in light of the broader long run deworming literature? In the main treatment effect specifications, much depends on the approach taken to treatment estimates which are positive but do not reach conventional levels of significance. In this case, the power of the study and the 95% confidence intervals of the estimated treatment effects should also be considered. The main regressions for numeracy have 95% confidence intervals of (-0.1, 0.24), meaning that the study lacks sufficient power to rule out large positive effects, as well as more modest negative effects. These wide confidence intervals are a function of the fact that this study took key design parameters (especially number of clusters) as fixed by the original trial and the subsequent Uwezo data collection exercise.

While p values above or below 0.05 are often interpreted in binary terms, this has long been criticized by epidemiologists and statisticians [50, 51]. In this “vote counting” paradigm, Baird et al. [7] and Ozier [6] count as evidence for the long run benefits of deworming (p<0.05) while the results presented here count as evidence against (p>0.05). A better approach would be to formally aggregate these results via meta-analysis. However, whereas short-run deworming trials have typically measured a small set of outcomes, such as height and weight, consistently across trials, making meta-analysis very feasible, comparable meta-analysis is more challenging for long run studies. The treatments differ (direct deworming versus exposure via spillovers), as do the populations of interest (all exposed cohorts versus children under age 1, as in [6]), and the primary outcomes studied differ (labor market outcomes [7] versus generalized cognitive ability [6] versus numeracy and literacy (this paper).

While the deworming treatments differ and the outcomes measured are not directly comparable, like Baird et al. [7] we find educational gains concentrated among females, and like Ozier [6] and Miguel and Kremer [5] we find suggestive evidence of very local spillovers. The smaller point estimates of treatment effect are also consistent with this literature, given that the worm burden in the Ugandan setting, while high in absolute terms, was notably lower than the burden in western Kenya where the original deworming trial (on which Ozier [6] and Baird et al. [7] are based) took place.

Relating this back to policy, a frequentist policymaker might dismiss the evidence from this setting as too imprecise to use. A more Bayesian-minded policymaker, with imprecise information about potential benefits, but needing to decide about a deworming program on the basis of this information, might compare the expected benefit of the policy to the expected cost [52]. This policymaker might be primarily concerned about the effect of deworming on the health of the STH-infected, but in a high prevalence setting, it also makes sense to consider potential long run benefits to learning. A Bayesian policymaker would not consider a point estimate that is statistically indistinguishable from zero as effectively a zero (evidence of no effect), but would consider it as the most likely of a distribution of potential effect sizes which also includes zero and negative numbers. Then the decisionmaker would compare the expected costs and benefits of this intervention to other policies to increase learning.

To give context for cost-effectiveness, McEwan [53] estimates cost effectiveness ratios for a subset of learning interventions. Most studies require between 10 USD and 100 USD (PPP adjusted) to generate 0.2 SD of learning gain. A version of Uganda’s Child Health Day program which only included Vitamin A supplementation and deworming cost $0.22 per child served, which includes both financial outlays, staff time, and in-kind contributions [54]. These costs as were 73% driven by the deworming component ($0.16 per recipient) and 27% by Vitamin A supplementation ($0.06 per recipient). Since CHDs are conducted two times per year, this implies $0.32 per child per year for the deworming component, or $0.96 total per child for a three year program like the one studied here. A cost of $0.96 for 0.07 SD for learning implies an expenditure of $2.74 per 0.2 SD of learning. Even allowing for imprecision or differential assumptions between the cost estimates from Uganda’s Child Health Day deworming [54] and the various education trials [53], these cost ratios are favorable to deworming. In conjunction with the policymaker’s prior views on the value of deworming and the local prevalence and intensity of worm infection, a policymaker who significantly discounted the probability that 0.07 SD was the true effect on numeracy might still choose to invest in preschool deworming [55].

More generally, how should these finding affect interpretation of the long run impact of deworming? Clearly, the estimated treatment effects here are smaller than the large effects estimated in the original working paper using 2010 and 2011 data, and are no longer statistically significant. The case for meaningful long run effects is therefore at least somewhat less convincing than it would be if the results of this study, like that of Baird et al. [7] and Ozier [6], still provided strong evidence of large effects of mass deworming.

Yet this study should be interpreted in the context of the serious challenges involved in generating evidence about the long run effects of interventions such as deworming. The ideal research design for long run effects involves well-powered trials followed by detailed longitudinal data collection. This is the model of the original Miguel and Kremer [5] trial and the Baird et al. [7] follow up, and studies stemming from this original experiment have generated all of the experimental evidence on the long run effect of deworming to date. Outside of this setting, and apart from conducting new trials and waiting a decade or more for new long-run results, another approach is to identify experimental interventions, such as the one on which this paper is based, for which existing data can be used to assess long run impact. The benefit of this approach is the limited marginal cost of the exercise, and the (relative) speed with which results can be generated. The downside is that in this type of research design, control over study parameters such as measurement, survey design, and statistical power are limited. Results should therefore be interpreted in this light.

## Supporting information

S1 Text Additional details about the Uwezo sample. (PDF)

S1 Table Main robustness checks. (PDF)

S2 Table Treatment effects by survey rounds, 2010-2015. (PDF)

S3 Table Analysis of non-response by treatment status and additional covariates. (PDF)

S4 Table Relationship between education and migration in 2009-2010 Uganda National Panel Survey. (PDF)

S5 Table School attendance and enrolment by treatment interactions. (PDF)

S6 Table Primary outcome regressions using survey weights. (PDF)

S7 Table Primary outcome regressions using imputed test scores and inverse probability weights to adjust for non-response. (PDF)
