# Supplementary material for: The long run impact of early childhood deworming on numeracy and literacy: Evidence from Uganda
Source: PLoS Negl Trop Dis. 2019 Jan 31;13(1):e0007085. doi: 10.1371/journal.pntd.0007085 (PMC6377149; doi:10.1371/journal.pntd.0007085)
Supplement: S1 Text — (PDF) [file pntd.0007085.s001.pdf]

Text S1: Additional details about the Uwezo sample

Uwezo enumeration areas were selected from the Ugandan census sampling frame using probability proportional to size, all households were then listed, and selected via systematic random sampling.

There was a partial village-level panel component from 2010–2012. While in each year, 30 villages were sampled per district, between 2010, 2011, and 2012 these were not separate random samples: 20 villages were re-sampled while 10 villages were newly sampled. There is no imbalance in the number of respondents sampled per village between treatment and control: a regression of treatment on the number of respondents per parish has a p value of 0.89 (unadjusted).

Since all students were sampled at home, and surveys took place during the school term, boarding school students were missed in all surveys.
