# Supplementary material for: The long run impact of early childhood deworming on numeracy and literacy: Evidence from Uganda
Source: PLoS Negl Trop Dis. 2019 Jan 31;13(1):e0007085. doi: 10.1371/journal.pntd.0007085 (PMC6377149; doi:10.1371/journal.pntd.0007085)
Supplement: S1 Table — (PDF) [file pntd.0007085.s002.pdf]

Table S1: Main robustness checks

	numeracy		literacy		total	
	(1)	(2)	(3)	(4)	(5)	(6)
**Panel A: water access and mother's education subsamples**						
treat	0.171**	0.109	0.125	0.0372	0.164*	0.0811
	(0.0839)	(0.119)	(0.107)	(0.121)	(0.0921)	(0.114)
N	1519	358	1524	361	1507	355
R2	0.268	0.389	0.272	0.352	0.309	0.407
**Panel B: water access and mother's education controls**						
treat	0.0670	0.0258	0.0427	0.0273	0.0620	0.0292
	(0.0834)	(0.0721)	(0.108)	(0.0997)	(0.0908)	(0.0777)
N	2052	1905	2053	1905	2031	1884
R2	0.231	0.252	0.247	0.264	0.274	0.297
**Panel C: Asset ownership and private schooling controls**						
treat	0.0741	0.0808	0.0625	0.0652	0.0758	0.0814
	(0.0799)	(0.0788)	(0.100)	(0.108)	(0.0846)	(0.0869)
N	2052	2010	2053	2009	2031	1990
R2	0.237	0.248	0.265	0.278	0.286	0.298
**Panel D: district fixed effects**						
treat	0.0607	0.0321	0.0609	0.0443	0.0627	0.0391
	(0.0611)	(0.0613)	(0.0696)	(0.0740)	(0.0574)	(0.0585)
N	2052	2052	2053	2053	2031	2031
R2	0.0346	0.259	0.0488	0.285	0.0446	0.310

These robustness checks follow the specifications used in the 2014 working paper discussed in the main article text. Panel A restricts the sample to respondents where households reports not having direct access to water access (odd numbered columns) or where the respondent's mother did not have any education (even columns). Panel B controls for these variables rather than restricting the sample. Panel C includes wealth quintiles controls in columns 1, 3, and 5, and controls for private schooling in columns 2, 4, and 6. Panel D includes district fixed effects in all columns. For panels A-D, additional controls (in columns 2, 4, and 6) include gender, age, and survey round, and all interactions of these variables. Robust standard errors clustered at parish level in parentheses.
* $p < .1$, ** $p < .05$, *** $p < .01$
