# Supplementary material for: The long run impact of early childhood deworming on numeracy and literacy: Evidence from Uganda
Source: PLoS Negl Trop Dis. 2019 Jan 31;13(1):e0007085. doi: 10.1371/journal.pntd.0007085 (PMC6377149; doi:10.1371/journal.pntd.0007085)
Supplement: S2 Table — (PDF) [file pntd.0007085.s003.pdf]

Table S2: Treatment effects by survey round

	numeracy		literacy		total	
	(1)	(2)	(3)	(4)	(5)	(6)
**Panel A: 2010-2011 sample**						
treat	0.326**	0.367**	0.159	0.223*	0.278	0.334**
	(0.156)	(0.140)	(0.171)	(0.127)	(0.165)	(0.131)
N	716	716	712	712	711	711
**Panel B: 2012 sample**						
treat	0.216	0.178	0.231	0.193	0.238	0.196
	(0.150)	(0.145)	(0.147)	(0.155)	(0.153)	(0.153)
N	540	540	539	539	539	539
**Panel C: 2013 sample**						
treat	-0.366*	-0.395*	-0.230	-0.279	-0.346**	-0.380**
	(0.188)	(0.188)	(0.180)	(0.187)	(0.159)	(0.165)
N	499	499	502	502	486	486
**Panel D: 2014 sample**						
treat	0.234	0.338*	0.219	0.277	0.236	0.327*
	(0.188)	(0.148)	(0.167)	(0.219)	(0.156)	(0.157)
N	106	106	107	107	106	106
**Panel E: 2015 sample**						
treat	-0.211*	-0.197	-0.215	-0.170	-0.223	-0.205
	(0.120)	(0.124)	(0.198)	(0.190)	(0.130)	(0.122)
N	191	191	193	193	189	189

Additional controls (in columns 2, 4, and 6) include gender, age, and survey round, and all interactions of these variables. Robust standard errors clustered at parish level.
* p< .1, ** p< .05, *** p< .01
