# Supplementary material for: The long run impact of early childhood deworming on numeracy and literacy: Evidence from Uganda
Source: PLoS Negl Trop Dis. 2019 Jan 31;13(1):e0007085. doi: 10.1371/journal.pntd.0007085 (PMC6377149; doi:10.1371/journal.pntd.0007085)
Supplement: S3 Table — (PDF) [file pntd.0007085.s004.pdf]

Table S3: Survey non-response

	(1)	(2)	(3)	(4)
treat	-0.0325	-0.0329	-0.0135	-0.0134
	(0.0295)	(0.0316)	(0.0223)	(0.0218)
age 9		0.0259	0.0336	0.0276
		(0.0249)	(0.0218)	(0.0202)
age 10		0.0231	0.0306	0.0271
		(0.0222)	(0.0205)	(0.0207)
age 11		-0.00373	0.00801	0.00359
		(0.0252)	(0.0230)	(0.0228)
age 12		0.0343	0.0399**	0.0366**
		(0.0204)	(0.0179)	(0.0176)
age 13		0.0353*	0.0420**	0.0383**
		(0.0183)	(0.0159)	(0.0161)
age 14		0.0345	0.0426*	0.0391*
		(0.0242)	(0.0224)	(0.0225)
age 15		0.0476**	0.0566***	0.0528***
		(0.0226)	(0.0185)	(0.0185)
age 16		0.110***	0.124***	0.120***
		(0.0360)	(0.0338)	(0.0336)
2011 rd		0.0107	0.0766*	0.0797*
		(0.0291)	(0.0453)	(0.0456)
2012 rd		0.0516	0.109**	0.111**
		(0.0423)	(0.0505)	(0.0522)
2013 rd		0.0280	0.0834	0.0859
		(0.0363)	(0.0508)	(0.0523)
2014 rd		-0.0231	0.0614	0.0629
		(0.0475)	(0.0545)	(0.0556)
2015 rd		-0.00683	0.0281	0.0292
		(0.0376)	(0.0546)	(0.0553)
female		-0.00783	-0.00892	-0.00929
		(0.0128)	(0.0123)	(0.0123)
district f.e.	no	no	yes	yes
wealth controls	no	no	no	yes
constant	0.0963***	0.0374	-0.00596	0.000257
	(0.0227)	(0.0313)	(0.0602)	(0.0529)
N	2210	2210	2210	2210

Dependent variable=1 if the respondent was present in household but did not participate in the assessment, resulting in missing value for numeracy or literacy score. Robust standard errors clustered at parish level in parentheses.
* p< .1, ** p< .05, *** p< .01
