# Supplementary material for: The long run impact of early childhood deworming on numeracy and literacy: Evidence from Uganda
Source: PLoS Negl Trop Dis. 2019 Jan 31;13(1):e0007085. doi: 10.1371/journal.pntd.0007085 (PMC6377149; doi:10.1371/journal.pntd.0007085)
Supplement: S4 Table — (PDF) [file pntd.0007085.s005.pdf]

Table S4: Correlation between literacy and migration

	(1)	(2)	(3)	(4)
**Panel A: full UNPS sample**				
can read/write	0.0415***	0.0284***	0.0324***	0.0242***
	(0.00933)	(0.00907)	(0.00847)	(0.00843)
age fixed effects	yes	yes	yes	yes
poverty controls	no	yes	yes	yes
demographic controls	no	no	yes	yes
district fixed effects	no	no	no	yes
Constant	0.821***	0.841***	0.822***	1.418***
	(0.0185)	(0.0187)	(0.0219)	(0.0240)
N	14811	14621	14621	14621
**Panel B: school age sample only (age 6-16)**				
literacy (0-3 scale)	0.0199***	0.0135***	0.00945**	0.00752*
	(0.00451)	(0.00447)	(0.00442)	(0.00454)
age fixed effects	yes	yes	yes	yes
poverty controls	no	yes	yes	yes
demographic controls	no	no	yes	yes
district fixed effects	no	no	no	yes
N	5017	5009	5009	5009

Data are from the 2009-2010 Uganda National Panel Survey. Dependent variable *any move* =1 if the household member spent at least 1 month away from home over the previous 12 months. Both panels show a linear probability model (LPM). The independent variable in Panel A is a binary indicator of whether the respondent can read and write; for Panel B it s a continuous (0-3) measure of literacy. Panel B is also restricted to respondents between age 6 and 16. Demographic controls are gender, whether the respondent is a nuclear member of the household head's family, and urban/rural location. Robust standard errors clustered at survey cluster level in parentheses.
* p< .1, ** p< .05, *** p< .01
