# Supplementary material for: The long run impact of early childhood deworming on numeracy and literacy: Evidence from Uganda
Source: PLoS Negl Trop Dis. 2019 Jan 31;13(1):e0007085. doi: 10.1371/journal.pntd.0007085 (PMC6377149; doi:10.1371/journal.pntd.0007085)
Supplement: S5 Table — (PDF) [file pntd.0007085.s006.pdf]

Table S5: Treatment interactions and school attendance

	(1) in school	(2) in school	(3) never enrolled	(4) never enrolled
treat × female	-0.0226	-0.0153	-0.00606	-0.00627
	(0.0145)	(0.0137)	(0.00679)	(0.00636)
treat	0.000777	-0.00663	0.0101*	0.0100
	(0.0135)	(0.0134)	(0.00593)	(0.00605)
female	0.0196**	0.0153	0.00198	0.00627
	(0.00956)	(0.0137)	(0.00191)	(0.00636)
treat × others treated	0.0146**	0.0156**	-0.00199	-0.00187
	(0.00686)	(0.00662)	(0.00181)	(0.00178)
treat	-0.0333*	-0.0384**	0.0102	0.00984
	(0.0187)	(0.0184)	(0.00634)	(0.00637)
others treated	0.00363	-0.0000401	-0.000225	0.000156
	(0.00278)	(0.00303)	(0.000259)	(0.000608)
treat × low assets	-0.0222	-0.0165	-0.00449	-0.00430
	(0.0135)	(0.0139)	(0.00826)	(0.00884)
treat	-0.000585	-0.00700	0.00901	0.00886
	(0.0132)	(0.0135)	(0.00775)	(0.00825)
low assets	0.0118	0.00764	-0.00446	-0.00476
	(0.00887)	(0.00799)	(0.00431)	(0.00455)
treat × under 14	-0.00523	-0.00206	0.00744	0.00852
	(0.0207)	(0.0215)	(0.00594)	(0.00597)
treat	-0.00735	-0.0129	0.00269	0.00192
	(0.0198)	(0.0202)	(0.00390)	(0.00344)
under14	0.0342**	0.00206	0.000917	-0.00852
	(0.0137)	(0.0215)	(0.000915)	(0.00597)
treat × 2−4 years exposure	0.0231	0.0124	-0.000988	0.00570
	(0.0249)	(0.0167)	(0.0227)	(0.0135)
treat	-0.0280	-0.0223	0.00691	0.0000203
	(0.0299)	(0.0191)	(0.0232)	(0.0144)
exposed 2-4 years	0.0622***	-0.0137	-0.0854***	-0.0383
	(0.0104)	(0.0772)	(0.0137)	(0.0335)
treat x program eligible at age 1	-0.0520**	-0.0452**	0.0110**	0.0101**
	(0.0209)	(0.0194)	(0.00504)	(0.00465)
eligible at age 1	0.0456***	0.234***	-0.00549*	0.000128
	(0.0158)	(0.0818)	(0.00284)	(0.00108)
treat	0.0248	0.0161	-0.0000235	0.000177
	(0.0168)	(0.0162)	(0.00379)	(0.00359)

Controls (in columns 2 and 4) include gender, age, and survey round, and all interactions of these variables.
Robust standard errors clustered at parish level in parentheses * p< .1, ** p< .05, *** p< .01
