# Supplementary material for: The long run impact of early childhood deworming on numeracy and literacy: Evidence from Uganda
Source: PLoS Negl Trop Dis. 2019 Jan 31;13(1):e0007085. doi: 10.1371/journal.pntd.0007085 (PMC6377149; doi:10.1371/journal.pntd.0007085)
Supplement: S6 Table — (PDF) [file pntd.0007085.s007.pdf]

Table S6: Main analysis using Uwezo survey weights

	numeracy		literacy		total	
	(1)	(2)	(3)	(4)	(5)	(6)
treat	0.0550	0.124	0.0152	0.0742	0.0419	0.114
	(0.102)	(0.0850)	(0.135)	(0.108)	(0.112)	(0.0881)
N	2052	2052	2053	2053	2031	2031

Survey weights provided by Uwezo are included in all regressions. Controls (in columns 2, 4, and 6) include gender, age, and survey round, and all interactions of these variables. Robust standard errors clustered at parish level in parentheses * p< .1, ** p< .05, *** p< .01
