# Supplementary material for: The long run impact of early childhood deworming on numeracy and literacy: Evidence from Uganda
Source: PLoS Negl Trop Dis. 2019 Jan 31;13(1):e0007085. doi: 10.1371/journal.pntd.0007085 (PMC6377149; doi:10.1371/journal.pntd.0007085)
Supplement: S7 Table — (PDF) [file pntd.0007085.s008.pdf]

Table S7: Main analysis with imputed outcomes and inverse probability weights

	numeracy		literacy		total	
	(1)	(2)	(3)	(4)	(5)	(6)
**Panel A: Using imputed outcomes**						
treat	0.0423	0.0638	0.0351	0.0536	0.0420	0.0636
	(0.0747)	(0.0748)	(0.107)	(0.0991)	(0.0867)	(0.0815)
N	2210	2210	2210	2210	2210	2210
**Panel B: Inverse probability weights for non-response**						
treat	0.0532	0.0682	0.0435	0.0535	0.0499	0.0652
	(0.0798)	(0.0814)	(0.114)	(0.105)	(0.0917)	(0.0879)
N	2052	2052	2053	2053	2031	2031

In Panel A, actual responses are used when available; in cases when no response was given by respondent, imputed values generated by Uwezo are substituted for missing values. In Panel B, inverse probability weights for non-response are generated through logistic regression of non-response with age, survey round, and gender included as predictors. Controls (in columns 2, 4, and 6) include gender, age, and survey round, and all interactions of these variables. Robust standard errors clustered at parish level in parentheses * $p < .1$, ** $p < .05$, *** $p < .01$
